# Forensic imaging

**DOI:** 10.1080/20961790.2017.1317691

**Published:** 2017-05-15

**Authors:** Silke Grabherr

**Affiliations:** University Centre of Legal Medicine Lausanne-Geneva, Lausanne, Switzerland

The use of images to explain and document findings for forensic and medico-legal purposes is as old as the discipline itself. Indeed, images seem an excellent complement to oral descriptions. Additionally, imaging techniques complete findings obtained today by conventional examinations as they have done since the very beginning of forensic medicine. Schemas of lesions or crime scenes were first drawn by the medical examiners. Later, photographs were used to document important medico-legal findings. A significant scientific advancement came with the discovery of X-rays by Konrad Roentgen, which then not only allowed experts to document the outside of a body, but also to observe and record the inside. Since this discovery, X-ray technology has continued to be used for forensic and anthropological purposes and has accompanied medico-legal investigations for more than a century.

During the last decade, modern imaging techniques have become more and more important in forensic and legal medicine. The development of rapid cross-sectional imaging techniques in the field of radiology has led to an explosion of new possibilities to rapidly examine the inside of a body. Modern computed software allows visualization of findings and tissues in two and three dimensions. New methodologies in the field of photography and 3D-modelling have also appeared and are becoming ever more straightforward and rapid to apply, allowing their use not only in the laboratory but at the crime scene as well.

However, the introduction of new techniques in the field of forensic medicine poses new questions. What are the limitations of these methods? Who should apply them and what kind of practice is needed to apply them correctly? What are the potential errors that can arise, and are the techniques really adequate for answering medico-legal questions? All these uncertainties can only be overcome by rigorous scientific exploration, which is the reason why efforts must be made to test new techniques and define both their advantages and weaknesses. Additionally, any technique can only be as good as the user applying it. Therefore, education and further training for forensic experts is necessary if we are to introduce new technologies successfully.

This special issue aims to assess the aforementioned questions and to inform the forensic community about existing techniques, actual developments and open questions. It gives a broad spectrum of information included in review articles, explains technical challenges through technical notes, and shows the application of methods in real cases using case reports. Open questions are also addressed in original studies. Methodologies described in this special issue cover the whole field of forensic imaging. Therefore, the described methods range from simple photography to modern cross-sectional imaging techniques such as Magnetic Resonance Imaging (MRI), Multi-Detector Computed Tomography (MDCT), minimally invasive imaging techniques such as Post-mortem Angiography and Image Guided Sampling, to finally conclude with high-resolution 3D modelling. The contributions from the authors also cover different fields of application, from legal medicine to anthropology and archaeology, a range of forensic sciences and even include discussion of some legal issues related to forensic imaging.

In view of this wide-ranging content, this issue may be an excellent tool to obtain an overview of forensic imaging or provide up-to-date information on actual research for those who are already in this field. It will bring the fascinating world of forensic imaging closer to the readers and give them an understanding of the medico-legal questions that can be solved by imaging, as well as the problems that remain to be addressed in this field.

